# UK Facioscapulohumeral Muscular Dystrophy (FSHD) Patient Registry

**DOI:** 10.1186/1750-1172-9-S1-P6

**Published:** 2014-11-11

**Authors:** Libby Wood, Teresinha Evangelista, Fiona Norwood, Richard Orrell, Marita Pohlschmidt, Mark Busby, Andrew Graham, David Hilton-Jones, Cheryl Longman, Peter Lunt, Mark Roberts, Stuart Watt, Suzanne Watt, Tracey Willis, Hanns Lochmüller

**Affiliations:** 1 Institute of Genetic Medicine, Newcastle University, Newcastle-upon-Tyne, UK; 2 King’s College Hospital, London, UK; 3 Institute of Neurology, University College London, London, UK; 4 Muscular Dystrophy Campaign, London, UK; 5 Bradford Royal Infirmary, Bradford, UK; 6 Division of Clinical Neurology, University of Oxford, Oxford, UK; 7 Southern General Hospital, Glasgow, UK; 8 National Genetics Education and Development Centre, Birmingham, UK; 9 Salford Royal, Manchester, UK; 10 The Robert Jones and Agnes Hunt Orthopaedic Hospital, Oswestry, UK

## 

The United Kingdom (UK) Facioscapulohumeral Dystrophy (FSHD) Patient Registry launched in May 2013. Funded by the Muscular Dystrophy Campaign and supported by the TREAT-NMD Alliance. This patient driven registry collects the internationally agreed core dataset, an outcome of an ENMC Workshop held in 2010 [1], through a novel online portal (http://www.fshd-registry.org/uk). Genetic details are added by a nominated neuromuscular specialist. In addition questionnaires about pain, quality of life and scapular fixation are included.

In the 12 months between May 2013 and May 2014 over 400 people registered, 92% with a diagnosis of FSHD1. Similar proportions of patients registered from both sexes and 59% of patients were between 40 and 70 years old (mean 47.39). Muscle weakness was widely reported with periscapular shoulder weakness occurring most frequently (89%) followed by weakness of the hip girdle (73%), facial muscles (72%) and foot dorsiflexor (71%). The onset of facial weakness was reported significantly earlier than weakness in other areas with 66% experiencing facial weakness before 20 years old.

Full time wheelchair use was reported in 18% of cases, 62% having lost ambulation between 31 and 60 years old (mean 41.61). Use of a wheelchair or other assistive device part time was reported in 44% of cases. A small proportion of patients report hearing loss (18%), retinal vascular disease (2%) and using ventilation (7%).

Additional questionnaires on pain were completed by 350 patients during this time and the majority reporting at least some pain, most often described as tiring or aching. Persistent pain (experienced for at least 3 months in a year) was reported by 92% with 53% of people describing this pain as distressing, horrible or excruciating. The location of the pain is variable but most often reported in the shoulder.

A broad spectrum of patients has registered providing a new insight into the FSHD population in the UK. The Registry aims to help facilitate and accelerate clinical research and trials, sharing a common dataset with a growing number of FSHD registries around the world will allow the registry to achieve this locally and internationally. The registry is well placed to inform future clinical research and help develop of standards of care.
